# Perspectives on the Molecular Mediators of Oxidative Stress and Antioxidant Strategies in the Context of Neuroprotection and Neurolongevity: An Extensive Review

**DOI:** 10.1155/2022/7743705

**Published:** 2022-08-26

**Authors:** Sheikh Shohag, Shomaya Akhter, Shahidul Islam, Tonmoy Sarker, Moinuddin Khan Sifat, Md. Mominur Rahman, Md. Rezaul Islam, Rohit Sharma

**Affiliations:** ^1^Department of Genetic Engineering and Biotechnology, Bangabandhu Sheikh Mujibur Rahman Maritime University, Dhaka, Bangladesh; ^2^Department of Biochemistry and Molecular Biology, Faculty of Life Science, Bangabandhu Sheikh Mujibur Rahman Science and Technology University, Gopalganj 8100, Bangladesh; ^3^Department of Pharmacy, Faculty of Allied Health Sciences, Daffodil International University, Dhaka 1207, Bangladesh; ^4^Department of Rasa Shastra and Bhaishajya Kalpana, Faculty of Ayurveda, Institute of Medical Sciences, Banaras Hindu University, 221005, Varanasi, Uttar Pradesh, India

## Abstract

Molecules with at least one unpaired electron in their outermost shell are known as free radicals. Free radical molecules are produced either within our bodies or by external sources such as ozone, cigarette smoking, X-rays, industrial chemicals, and air pollution. Disruption of normal cellular homeostasis by redox signaling may result in cardiovascular, neurodegenerative diseases and cancer. Although ROS (reactive oxygen species) are formed in the GI tract, little is known about how they contribute to pathophysiology and disease etiology. When reactive oxygen species and antioxidants are in imbalance in our bodies, they can cause cell structure damage, neurodegenerative diseases, diabetes, hypercholesterolemia, atherosclerosis, cancer, cardiovascular diseases, metabolic disorders, and other obesity-related disorders, as well as protein misfolding, mitochondrial dysfunction, glial cell activation, and subsequent cellular apoptosis. Neuron cells are gradually destroyed in neurodegenerative diseases. The production of inappropriately aggregated proteins is strongly linked to oxidative stress. This review's goal is to provide as much information as possible about the numerous neurodegenerative illnesses linked to oxidative stress. The possibilities of multimodal and neuroprotective therapy in human illness, using already accessible medications and demonstrating neuroprotective promise in animal models, are highlighted. Neuroprotection and neurolongevity may improve from the use of bioactive substances from medicinal herbs like *Allium stadium*, *Celastrus paniculatus*, and *Centella asiatica.* Many neuroprotective drugs' possible role has been addressed. Preventing neuroinflammation has been demonstrated in several animal models.

## 1. Introduction

A disruption in the balance of reactive oxygen species such as superoxide, hydroxy radicals, and nitric oxide radicals, as well as antioxidants such as vitamins A, C, and E, selenium, and carotenoids, which can cause tissue damage, is known as oxidative stress. In biological systems, oxidative stress is spread as metabolic by-products [1, 2]. It can come from both endogenous (in mitochondria during oxidative phosphorylation, during inflammation, the respiratory burst, endoplasmic reticulum, phagocytic cells, and peroxisomes for example) and exogenous (pollution, alcohol, tobacco smoke, heavy metals, transition metals, industrial solvents, pesticides, certain drugs like paracetamol, and UV radiation, halothane, and exogenous sources) [3]. When ROS and RNS are produced in our bodies at moderate or low levels, they help the heart pump more blood in stressful situations and also perform a variety of physiological functions such as immune function and a variety of cellular signaling pathways in the redox regulation pathway; nitric oxide (NO°) helps to regulate blood pressure during mitogenic responses, and phagocytic cells use them to kill bacteria during bacterial infections [4, 5]. Exogenously or endogenously produced highly reactive oxidizing chemicals are always a threat to living organisms. They can take electrons from oxidizing compounds with ease. This results in cell structural damage, diabetes, hypercholesterolemia, atherosclerosis, cancer, cardiovascular diseases, metabolic disorders, and other obesity-related problems [6–9]. When a person is under oxidative stress, free radicals assault his neural cells, putting them at risk of degeneration. The cytotoxic effect of ROS can cause protein misfolding, mitochondrial malfunction, glial cell activation, and subsequent cellular apoptosis [10].

There are two terms in “neurodegeneration”: “neuro” represents for nerve cells, and “degeneration” means for deterioration. The loss of neuron function is a characteristic feature of neurodegenerative disorders [11–13]. Degenerative nerve illnesses can affect a person's mobility, vision, memory, IQ, speech, respiration, heart function, and much more. The gradual loss of the structure or functionality of neurons, or neurodegeneration, is the root cause of neurodegenerative diseases. From the molecular to the systemic levels of neural circuitry, neurodegeneration may be seen in the brain. Alzheimer's disease and associated memory disorders ataxia, multiple system atrophy, Huntington's disease, motor neuron disease, Parkinson's disease, progressive supranuclear palsy, and amyotrophic lateral sclerosis are all examples of neurodegenerative disorders [14]. There is no proven treatment for Alzheimer's disease (AD) [15]. Phytochemicals have been shown to be beneficial in the treatment of neurodegenerative illnesses including Alzheimer's and Parkinson's disease (PD). Traditional herbs and phytochemicals may delay its development and decrease its course and enable healing by addressing several pathogenic reasons. They control mitochondrial stress, apoptosis, free radical scavenging, and neurotrophic factors. The most prevalent neurodegenerative disorders include amyloidoses, tauopathies, alpha-synucleinopathies, and TDP-43 proteinopathies [16]. Many of these disorders are inherited, but they can also be caused by toxins, chemicals, or viruses. When neurodegenerative disorders are inherited in an autosomal dominant pattern, they have a 50% chance of recurrence [17]. Vitamin C (ascorbic acid) is an important antioxidant molecule in the brain because it aids in the preservation of the integrity and function of numerous brain processes [18]. However, an abnormally high vitamin C concentration can cause neurological problems. Many investigations have shown that preterm babies' neurological damage is caused by impairment of vitamin C transfer [19, 20]. Mortality from presenile dementia (PSD), Alzheimer's disease (AD), Parkinson's disease (PD), and motor neuron disease (MND) was examined for 27 states in national occupation mortality surveillance (NOMS) systems from 1982 to 1991, totaling 130,420 deaths, according to the CDC (Centers for Disease Control and Prevention) [21].

Oxidative stress is caused by an imbalance between the amount of reactive oxygen species (ROS) in the body and the ability of a biological system to quickly get rid of the reactive intermediates or fix the damage they cause. When the normal redox state of a cell is upset, free radicals and peroxides are made that damage proteins, lipids, and DNA [22]. Damage to DNA's bases and breaks in its strands are caused by oxidative stress, which comes from oxidative metabolism. The development of chronic fatigue syndrome (ME/CFS), cancer, Parkinson's disease, Lafora disease, Alzheimer's disease, atherosclerosis, heart failure, myocardial infarction, fragile X syndrome, sickle-cell disease [23], vitiligo, lichen planus, and ADHD [24] are all believed to be influenced by oxidative stress in humans. However, ROS can also be helpful because they are employed by the immune system to attack and kill pathogens.

Around 5 million Americans endure Alzheimer's disease, 1 million from Parkinson's disease, 400000 from multiple sclerosis (MS), 30,000 from amyotrophic lateral sclerosis (ALS), and 3000 from Huntington's disease in contemporary times (HD) [25]. Neuron cells have been found to be protected by a variety of phytochemicals. The purpose of this research covers oxidative stress and its link to neurodegenerative diseases, and thus a number of neuroprotective drugs along with several phytochemicals that have recently been shown to have neuroprotective effects in a lot of species.

## 2. Neurological Abnormalities and Oxidative Stress

Free radicals are reactive chemicals that are spontaneously created in the human body. They have either beneficial or detrimental effects on the immune system. An antioxidant system is required to reduce these adverse effects on an organism. When there is an imbalance between the production of reactive oxygen species and the antioxidant defense, a situation known as oxidative stress may be described. It is more difficult to define an antioxidant. Halliwell proposed a popular definition: Low concentrations of an antioxidant inhibit or significantly reduce the oxidation of an oxygen-soluble compound. Antioxidants are used to protect off oxidative damage. It is essential to mention that reducing agents are not the same as antioxidants, because they use different chemical words to explain the same activity. A lowering agent could even be used. If it converts transition metal ions to free radicals or reduces oxygen to free radicals, it is a prooxidant. Peroxides react more rapidly with lower oxidation states. Several biological lowering agents Janus-faced agents: Depending on the amounts of O_2_, they can be antioxidants or prooxidants [26].

Oxidants are reactive molecules that are formed both within the body and in the environment. These molecules have the potential to react with other biological components found in the body, including protein, DNA, and lipids. Several biological processes, such as aging, cancer, atherosclerosis, and dementia, are associated with tissue damage induced by oxidative stress and mediated by excessive free radicals. The genesis of oxidative stress is an imbalance between the creation of reactive oxygen/nitrogen species and the antioxidant capabilities of cells and organs. Reactive oxygen species (ROS) consist of superoxide anion (O2-), hydroxyl radicals (.OH), and hydrogen peroxide (H2O2), while antioxidants consist of numerous vitamins and endogenous enzymes, such as superoxide dismutase (SOD), catalase, and glutathione peroxidase [27]. Endogenous or exogenous reactive oxygen species can exist mitochondrial electron transport chain that is the predominant endogenous ROS emitter. The reduction of O_2_ to H_2_O_2_ occurs in four phases each of which produces ROS [28].

1. Superoxide radical O_2_ + e O_2_°
2. Hydroperoxyl radical O_2_° + H_2_O H_2_O°
3. Hydrogen peroxide (H_2_O° + e + HH_2_O_2_)
4. Hydroxyl radical H_2_O_2_ + H + OH°

Mitochondrial-targeted neuroprotective therapeutics mitochondria are a significant source of reactive oxygen species (ROS) in the central nervous system. They have redox carriers that may transport single electrons to oxygen, resulting in the production of ROS superoxide (O_2_). The tricarboxylic acid cycle (complexes I, II, and III) and electron transport chain (complexes I, II, and III) enzymes, as well as monoamine oxidases, are among the mitochondrial redox carriers that generate superoxide. Other production of reactive oxygen species enzymes can also be found in mitochondria. Superoxide is depleted and converted into hydrogen peroxide during a dismutation reaction by superoxide dismutase (SOD) (H_2_O_2_). To remove H_2_O_2_ from mitochondria, SOD enzymes cooperate with catalases and glutathione peroxidases. Nevertheless, O_2_ and H_2_O_2_ can generate hydroxyl radicals and peroxynitrites when those who react with other molecules in the cell, such as redox-active metals (Fenton reaction involving iron) and nitric oxide. These chemical processes occur a precise balance of Ros generation and eradication under standard circumstances. This balance is thrown off by aging or Alzheimer's disease increasing reactive oxygen species (ROS) and oxidative damage. Elevated numbers of mutations in mitochondrial DNA, as well as increased quantities of 8-hydroxy-2-deoxyguanosine, a hallmark of oxidative DNA damage, has been identified in AD [29, 30]. Mitochondrial dysfunction and apoptosis can result from either of these deletions or point mutations, which can be exacerbated by oxidative stress [31]. Several mitochondrial essential enzymes involved in ROS detoxification are also impacted in addition to DNA damage. The ketoglutarate dehydrogenase complex (KGDHC) [32], pyruvate dehydrogenase complex (PDHC), and cytochrome oxidase (COX) [33] are all significantly reduced in adult AD brains. In animal models, mitochondria have also been connected to the etiology of Alzheimer's disease.

## 3. Neuroregeneration in Neurodegenerative Disorders

**Figure 1:**
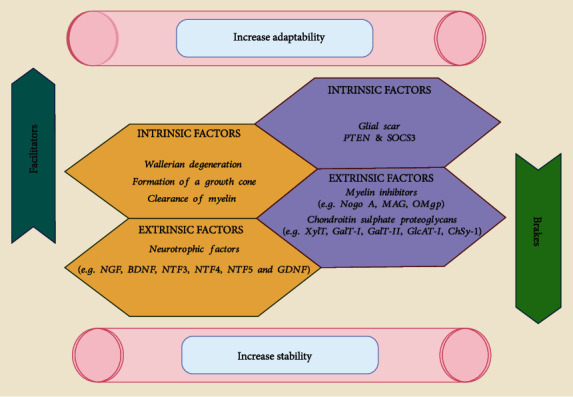
Central and peripheral nervous system neuroregeneration is influenced by extrinsic and intrinsic factors. For instance, the suppressor of cytokine signaling; PTEN (phosphatase and tensin homolog); NGF (nerve growth factor); SOCS3; MAG (myelin glycoprotein); keratin sulfate proteoglycans; myelin-associated glycoprotein; chondroitin sulfate proteoglycans; and oligodendrocyte [36].

The term “neuroregeneration” refers to generating new neurons, glial cells, axons, synapses, or myelin to heal or regenerate damaged neural tissue [26]. The term “neurodegeneration” is nonspecific. Degeneration can range from severe neuronal death and brain atrophy, as in late-stage AD, to degeneration in tiny neuronal structures, such as dendrites, spines, and axons, without neuronal death [34]. The central nervous system is mostly incapable of self-healing and regeneration, but the peripheral nervous system can. For all time, there has been no cure for CNS damage. There were no effective CNS regeneration therapy regimen available, and repeated efforts at neural re-growth failed owing to a lack of information regarding CNS regeneration. The discovery that mature neurons in the CNS may recover after injury has just put an end to this therapeutic nihilism [35]. Unlike PNS injuries, CNS injuries have poor prognoses due to their inability to repair neurons. This disorder may be caused by the human CNS's more complicated neural networks than other species [36]. By adding neurons to already complex brain networks, one runs the danger of producing system confusion, which is analogous to accidentally short-circuiting electrical equipment, which in turn raises the chance of having seizures. With illness or injury, these restrictions become obstacles to rehabilitation (Figure 1) [31, 32].

The capacity for axonal regeneration is mostly determined by inhibitory components of the environment that are extrinsic as well as the inherent regenerative potential of neurons. In wounded adult CNS neurons, the intrinsic neural pathways launching a growth program are similarly quite restricted [32, 33]. Axons quickly regenerate after damage to a peripheral nerve (PNS). Wallerian degeneration occurs in the axon's distal part, which is not attached to the cell body. The axon is disintegrated and fragmented as a consequence of this dynamic process. Glial cells, mostly macrophages, clear debris from the brain. It is therefore possible to rejuvenate and regenerate their targets so that function may be restored [37].

## 4. Neuroplasticity in Neurodegeneration Disorders

The ability of the brain to change through time is referred to as neuroplasticity. Adaptive behaviors, learning, and memory are at the top of the neuroplasticity hierarchy. Neuroplasticity couples functional changes with structural changes [38]. During ontogeny, phylogenesis, physiological learning, and brain damage, nervous system plasticity improves neuronal networks [36]. The molecular and cellular levels are both involved in the process of neuroplasticity, which manifests itself as short-term (STP), long-term (LTP), and long-term potentiation depression (LTD) [39]. Neuroplasticity is classified into two categories such as functional plasticity and structural plasticity. Learning and memory are the two fundamental mechanisms that underlie functional neuroplasticity. This sort of neural and synaptic plasticity is based on particular types of synaptic plasticity that cause persistent changes in synaptic efficacy. The synaptic connections between neurons change permanently throughout learning and memory as a result of structural alterations or intracellular metabolic activities [40]. The brain's capacity to modify neural connections is called structural plasticity. Neuroplasticity produces and integrates new neurons into the CNS during life. Researchers employ cross-sectional imaging (MRI, CT) to analyze structural brain changes [41]. Neuroplasticity investigates the influence of internal or external stimuli on brain remodeling. Structural neuroplasticity includes changes in grey matter percentage or synaptic strength. Current neuroscience research focuses on structural neuroplasticity [42]. The neuroplasticity hypothesis explains why individuals who have had a brain injury or have had a CVA recover abnormally [43]. Brain alterations are commonly seen as a sign of progress, although this is not necessarily true. There are several ways in which the brain's structure and function may be influenced or changed. A common illustration of how the brain's adaptability may be a problem is when drug abuse, sickness, or trauma leads to undesirable alterations (Involving damage to the brain or stressful events that lead to PTSD). Lead poisoning may impair your brain's capacity to adapt. Several medical problems may potentially restrict or inhibit brain plasticity [44]. As a result, children may be affected by a variety of pediatric neurological diseases, such as epilepsy and cerebral palsy [45]. Neuroplasticity may be guided to restore function and cure undesired symptoms in clinical settings by using a variety of therapeutic approaches [46]. Constraint-induced movement therapy (CIMT) is a type of physical therapy that has been studied a lot. Patients who have had a stroke may benefit from this technique because it forces the afflicted limb to perform repeated tasks and develop new behaviors. Contralateral premotor and secondary somatosensory cortex activity in the brains of patients who undergo this treatment has been shown to rise in correlation with better function [47]. Researchers have spent a lot of time and money studying how environmental influences might influence neuroplasticity. Studies have demonstrated that music therapy may have a favorable impact on neuroplasticity. Cognition and other executive functions have been proven to benefit from it. Researchers are looking at a variety of dietary supplements to see whether or not they might assist promote neuroplasticity [48].

## 5. Cellular and Molecular Immune Mediators of Neuroprotection

A neuroprotection system or approach aims to prevent the nervous system from being damaged or injured, especially in those who have been injured or diagnosed with a neurological condition [49]. The purpose of neuroprotection is to protect the central nervous system against early degeneration and other factors that may lead to the loss of nerve cells, as well as to limit the amount of nerve death [50]. It would seem that the central nervous system and the immune system (CNS) have developed a diverse set of mechanisms throughout the course of evolution in order to fight infections and react to stress. This is because the CNS immune response has a large number of failsafe mechanisms that facilitate a well-regulated response to injury and the commencement of healing and repair [51]. Neurotrophins are important regulators of neural growth function, [52] survival, and the ability to change (plasticity) [53]. Nerve growth factor, often known as NGF, is a neurotrophic factor that, in mammals like humans, helps to foster the development of sympathetic nerve cells and peripheral sensory, and ensures that they are able to survive. It was identified in 1950 [54]. NGF functions by activating two transmembrane receptors. Two examples are the trkA and p75 receptors, both members of the tropomyosin receptor kinase (trk) family [55].

Exosomes are important mediators of neurodegenerative disorders, carrying beta amyloid and prions from their source cells to other cells [56]. Exosomes may regulate neuroinflammation, enhance neurogenesis and neurogenic physiological location, and cure neurological disorders [57]. It has been shown that the CREB (cAMP-responsive element-binding protein) pathway is involved in the control of neuronal function. This is achieved by its involvement in two major cascades of gene expression. The first one explains that CREB is an important component of the molecular switch that controls more long-term forms of brain plasticity and learning. The second one connects CREB to the maintenance and protection of neuronal survival [58]. CREB is involved in a wide number of cellular functions, some of the most significant of which include cell differentiation, proliferation, metabolism, and survival [59].

## 6. Neuroprotective Agents and their Functions

Trying to protect the nervous system from neuronal loss and neurodegeneration can be achieved by using capable of differentiating to suppress pathophysiological pathways that can ultimately lead to damage to the nervous system (Table 1) [60]. Immunosuppressive calcineurin inhibitors are responsible such as NOS inhibitor, ca2+ channel blocker, cationic arginine-rich peptides, benzoic acid-derived nitrones, edaravone, AMPA antagonist cyclosporine A, and sulfur-containing secondary metabolites [61–63] . Neuronal cell death prevention was shown to be beneficial for several drugs in animal models, but human therapeutic studies have yet to confirm these finding Here, we will take a look at several promising neuroprotective drugs such as magnesium sulfate, statins, melatonin, erythropoietin, free radical scavengers, immunosuppressant drugs, N-acetyl-L-cysteine (NAC), *β*-blockers, COX-2 selective inhibitors, and curcumin that might be useful for patients in the intensive care unit [64].

### 6.1. Glutamate Blockers

The amino acid glutamate is the most prevalent in the brain. Glutamate has an increasing contribution on nerve cells even though glutamate receptors are present on some of these cells. It can cause cells to die. Glutamate activates several metabotropic receptors as well as three important ionotropic receptors: To avoid excitotoxicity, kainite amino-3-hydroxy-5-methyl-4-isoxazole propionic acid (AMPA), and N-methyl-D-aspartate (NMDA), glutamate blockers reduce NMDA and AMPA [65].

Glutamate transporters are a type of neurotransmitter transporter that removes extracellular glutamate to prevent excitotoxicity in neurons when glutamate tries to enter the synaptic cleft, the excitatory amino acid transporter (EAAT) family involves removing it, and when it wants to enter the cell cytoplasm, it required to transport it into synaptic vesicles. When EAAT 2 (human glutamate transporter 2) is not operating, it can cause traumatic brain damage, stroke, amyotrophic lateral sclerosis, and Alzheimer's disease. Some of the most widely accepted stroke professionals in rodents while also nonhuman primates and humans were using the glutamate blockers polyarginine R18 and NA-1 (TAT-NR2B9C). It lowers mitochondrial oxidative stress in neurons [66].

### 6.2. Statins

Statins are the first-choice therapeutic medicines for the prevention of cardiovascular disease (CVD) and atherosclerotic diseases due to high levels of cholesterol in the blood [84]. A growing body of research indicates that statins have other pleiotropic effects in addition to their vascular effects, such as stability of atherosclerotic plaques and reduced carotid intimal medial thickness, which are unrelated to their cholesterol-lowering impact [85]. These activities include decreasing the thickness of the carotid intimal medial layer. On the other hand, it is common knowledge that both theoretical and empirical research have shown that inflammation plays a crucial part in the mediation of every stage of atherosclerotic illnesses. In addition to having antioxidant, anti-inflammatory, and anti-platelet actions, statins also have a role in the protection of endothelial cells by acting on the enzyme that produces nitric oxide [86]. These effects of statins might have significant therapeutic significance in the treatment of a wide variety of neurological illnesses. There is an expanding body of research that points to a connection between neurodegenerative disorders and vascular risk factors including atherosclerosis; nevertheless, this connection is still considered to be speculative. In this study, we highlight and discuss the current state of knowledge regarding the effects of statins in stroke, Alzheimer's disease, Parkinson's disease, multiple sclerosis, and primary brain tumors. In addition, we report the potential adverse effects of statins as well as the restrictions placed on the use of these drugs [87].

Research on the effectiveness of statins as treatments for Alzheimer's disease and stroke is advancing at a breakneck pace. There is a wealth of evidence to support the use of these medicines in the pretreatment of ischemic stroke as well as in patients who have a history of cerebrovascular illness [88]. In the acute period of stroke, the use of statins for their brain-protective impact is being studied, along with many other possible therapies.

### 6.3. *Withania somnifera* (Ashwagandha)

One of the most expensive herbal medicines used in Indian traditional medicine (Ayurveda) is Ashwagandha, which is derived from the roots of the *Withania somnifera* Dunal plant and is used as a Rasayana medication to promote long life, young energy, and strong mental faculties [89]. Clinical studies have shown that Ashwagandha may cure a variety of conditions, including overall sluggishness, consumption, nervous weariness, sleeplessness, memory loss, and more [90]. By virtue of its historical applications, Ashwagandha could be able to treat neurodegenerative disorders. In fact, this herbal medication has been shown to have a variety of pharmacological actions, including those that are anti-inflammatory, antitumor, antioxidant, immunomodulatory, and antineuropsychiatric illness effects [91]. It is anticipated that therapeutic applications of Ashwagandha and its ingredients may result in improvements in neurodegenerative illnesses due to the plant's actions against these conditions [92]. Some organizations have in the past stated that Ashwagandha and its components are safe to use. In rats, giving them 100 milligrams per kilogram per day of an Ashwagandha water extract along with their drinking water for a period of eight months did not produce any harmful effects. The results of repeated oral administration of a methanol extract of Ashwagandha containing 80% (2000 mg/kg/day for 28 days) revealed no signs of toxicity [93]. However, acute toxicity was caused in mice when the alcoholic extract from the defatted seeds of Ashwagandha was given to them by oral administration; the LD50 value was 1750 41 mg/kg. After intraperitoneal injection of an ethanol extract of Ashwagandha, the LD50 in mice was determined to be 1259 mg/kg [94].

## 7. Neuroprotective Roles of Phytochemicals

Phytochemicals, from the Greek “Phyto” meaning “plant,” are chemical substances produced by plants. That phytochemical is used by plants to defend themselves against microbes [95]. Plants employ phytochemicals to protect themselves not only against microbes, but also from environmental threats like pollution, stress, and UV exposure [66]. It is responsible for the colors, perfume, and flavor of plants.

Antifungal, chemopreventive, anti-inflammatory hepatoprotective, hypolipidemic, neuroprotective, hypolipidemic, and hypotensive properties are all antifungal, antiallergenic, anti-inflammatory, antiallergenic, antispasmodic, hypolipidemic, and hypotensive properties of phytochemicals [96].

More than 4500 phytochemicals have been identified, although only 350 have been thoroughly investigated [41]. Protective properties, as well as physical and chemical features, are used to classify phytochemicals. More than 120 traditional remedies have been used in Asian countries to treat central nervous system disorders [97].

Over antiquity, medicinal plants have provided an assortment of bioactive components that support in the health and quite well of humans [98]. When used as a neuroprotective agent, they also have few adverse effects [41]. Plants produce bioactive chemicals as secondary metabolites. On humans and animals, it has pharmacological or toxicological effects. Bioactive chemicals have health-promoting effects on the body. They are being researched for cancer, heart disease, and other disorders prevention. Lycopene, lignan, tennis, indoles, terpenoids, glycosides, alkaloids, flavonoids, phenolic compounds, and other bioactive chemicals are examples. Plants and some foods, such like fruits, vegetables, nuts, and whole grains, contain modest levels of this substance. Carotenoids, choline, flavonoids, carnitine, coenzyme Q, dithiolthiones, phytosterols, phytosterols, glucosinolates, certain vitamins and minerals, polyphenols, tocotrienols, organosulfur compounds including isothiocyanates, lycopene, lignan, tennis, indoles, terpenoids, glycosides, and alkaloids are also included.

Medicinal plants are high in phytochemicals and antioxidants, which may assist to control the disease by reducing the progression of symptoms and problems while having few or no adverse effects [99]. About 80% of people in poor nations rely on primary healthcare for men and cattle [47]. Some medicinal plants and their bioactive compounds for neuroprotection are depicted in Table 2 [100].

### 7.1. *Bacopa monnieri*

Phytochemical *Bacopa monnieri*, rather than Brahmi, has neuroprotective properties [101]. It has been widely utilized in neuro medicine to treat a variety of ailments, including anxiety, depression, and memory loss. It possesses antioxidant, adaptogenic, and memory-enhancing properties. Bacoside A is a Bacoside chemical molecule with neuropharmacological effects. Bascopaside III, bacopaside X, bascoside A3, and bacopasaponin make up this chemical molecule. As a result, passive diffusion over the blood-brain barrier is accomplished by the non-polar glycosidic structure of A.

Several studies have shown that backside A, a bioactive component, protects the brain from oxidative damage and age-related cognitive decline through a variety of methods [102]. As illustrated by a high-resolution liquid chromatography (HPLC) study, it reduces Abeta aggregation and fibril formation that could interact directly or indirectly with neurotransmitter systems to improve memory and learning potential.

### 7.2. Allium Stadium

*Allium stadium* is renowned as “garlic” by the general public. You can get it in a range of methods, and it is a well-known and valuable spice [103]. Antioxidant, hypotensive, antimicrobial, antifungal, antitumorigenic, immunomodulatory, anti-inflammatory, hepatoprotective, anthelmintic, anticoagulant, and fibrinolytic characteristics are all present in it [104–107]. Garlic is high in potassium, phosphorus, zinc, sulfur (at least 30 sulfur-containing compounds including alliin, allyl propyl disulfide, vitamin A, C, and B complex, allicin dats, s-allyl cysteine, vinyldithiins, same), and myrosinase and low in sodium, ajoene, and various types of enzymes such as alliance and peroxidase. Garlic consumption has numerous health benefits due to its therapeutic properties against cancer, diabetes, hyperlipidemia, lowering blood pressure, bone and skin diseases, Parkinson disease, atherosclerosis, type 2 diabetes, and aged garlic extract, and its components exert neuroprotective effects in Alzheimer's disease, cardiovascular disease, Huntington disease, and cerebral ischemia models [108]. Garlic extract has been shown to preserve dopaminergic neurons in Parkinson's disease research. Oxidative stress, inflammation, and mitochondrial malfunction have also been prevented by the meal replacement, along with cell death. Adult male Wistar rats were given AGE (aged garlic extract) in doses of 125, 250, and 500 mg/kg, respectively, based on their body weight. It was repeated every day for 56 days. They were subsequently given a bilateral injection of 1 l of aggregated A (- amyloid) into the lateral ventricles. An NUR test seven days apparently showed that AGE dosages of 250 mg and 500 mg/kg BW significantly improved short-term recognition memory in cognitively impaired rats and also reduced the inflammatory response by minimizing microglia activation (-amyloid) in the cerebral hemispheres. In cognitively challenged rats, AGE dosages of 250 mg and 500 mg/kg BW drastically enhanced short-term recognition memory and also reduced the inflammation response by lowering microglia activation seven days later [109].

## 8. Potential Role of Antioxidant Vitamins and Synthetic Compounds for Neuroprotection

**Figure 2:**
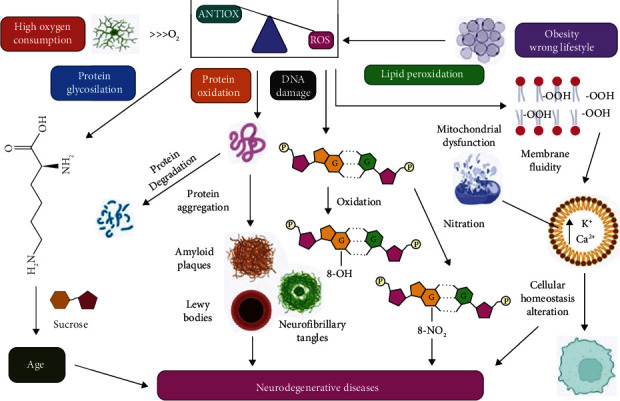
Oxidative stress hypothesis as well as its effects on a cellular level. What happens to free radicals when they are formed in cells? Because of the brain's high oxygen demand, ROS (reactive oxygen species) are constantly being produced (ROS). Since their high reaction rate increases oxidative stress and thus the formation of AGE and/or protein function loss, they also cause (i) protein oxidation and glycosylation, which leads to protein degradation; (ii) cell peroxidation, which reduces membrane fluidity and increases cellular permeability, which alters homeostasis in cells, and neurodegenerative diseases may be caused by any of these; and (iii) reasons DNA damage through guanine nucleotide oxidation or reduction [127].

The breakdown of the equilibrium between pro-oxidant and antioxidant owing to an excessive buildup of reactive oxygen species is known as oxidative stress (ROS). The central nervous system is especially vulnerable to ROS due to its high energy demand and metabolic rate, as well as an insufficient antioxidant defense mechanism and reduced ability for cellular regeneration [119]. An excessive amount of ROS may result in significant pathological damage. These pathological damages include inflammation, cell cycle regulation, stressor responses, enzyme and receptor activation, phagocytosis, more signal transduction, and gene expression. ROS, when present at quantities that are not excessive, are essential for the proper functioning of a number of physiological processes, such as signal transduction and gene expression [120]. Specifically, ROS oxidize polyunsaturated fatty acids, which are key biological targets. Nucleic acids are another potential biological target of free radicals. Ascorbate, commonly known as AA (ascorbic acid), is a potent antioxidant that is water-soluble and serves as a cofactor for a variety of enzymes. It is one of the most prevalent antioxidants [121, 122]. It is able to prevent the production of reactive oxygen species (ROS), directly remove ROS and RNS from the environment, and restore the functionality of other scavengers that have been damaged by oxidation. The AA concentration in striatal extracellular fluid was lowered in a transgenic HD mouse model; therefore, high dosages of ascorbate were used to restore the mice's normal behavior [123]. Tocopherols and tocotrienols are the two main classes of lipid-soluble antioxidants that make up vitamin E [124]. Among them, alpha-tocopherol is the type of vitamin E that exhibits the highest level of biological activity [125]. It is an antioxidant that can break chains while maintaining a low molar ratio in comparison to unsaturated phospholipids. Vitamin E protects cellular membranes from oxygen free radicals produced by polyunsaturated fatty acids and scavenges superoxide and hydroxyl radicals. Vitamin E recycling by vitamin C, ubiquinols, and thiols restores its antioxidant activity (Figure 2) [126].

### 8.1. Cellular and Molecular Immune Mediators of Neuroprotection

A neuroprotection system or technique aims to prevent the nervous system from being damaged or injured, especially in people who have been injured or diagnosed with a degenerative disease [128]. Neuroprotection aims to reduce nerve death following a CNS injury and to protect the CNS from early degeneration and other causes of nerve cell loss [50]. In order to beat infections and cope with stress, the immune system and the CNS appear to have evolved a wide and varied set of mechanisms. This is because the CNS immune response has a large number of failsafe mechanisms that facilitate a well-regulated response to injury and the commencement of healing and repair [51]. Neurotrophin**s** are important regulators of neural growth function, survival, and the ability to change (plasticity) [122, 129]. Nerve growth factor (NGF) is a neurotrophic factor that promotes the development and survival of peripheral sensory and sympathetic nerve cells in mammals, including humans. It was identified in 1950 [54]. NGF functions by activating two transmembrane receptors. Another one is the p75 receptor, which actually applies to the tropomyosin receptor kinase (trk) family [130]. Exosome**s** are important mediators of neurodegenerative disorders, carrying beta amyloid and prions from their source cells to other cells [56]. Exosomes may regulate neuroinflammation, enhance neurogenesis and neurogenic physiological location, and cure neurological disorders [131]. Gene expression is regulated by the cAMP-responsive element-binding protein (CREB) pathway, which participates in two key gene expression cascades. The first one describes CREB as an essential part of the molecular switch that regulates more permanent kinds of neural plasticity and learning. The second one connects CREB to the maintenance and protection of neuronal survival [58]. CREB plays an important role in a variety of cell processes, metabolism, including proliferation, differentiation, and survival [59].

## 9. Turmeric and Choline in Diet Can Increase the Neuroplasticity

### 9.1. Turmeric

In Southeast Asia, turmeric is widely used as a spice. It contains numerous health benefits, particularly in the cases of Alzheimer's disease and Parkinson's disease. Turmeric contains polyphenolic curcuminoids, diferuloylmethane, bisdemethoxycurcumin, dimethoxy curcumin, and cyclocurcumin, among other beneficial compounds. Curcumin contains numerous important physiological effects, anti-rheumatoid, including anti-inflammatory, antispasmodic, anti-allergy, and anticancer characteristics [132–134]. Curcumin activated extracellular signals, which regulated neuronal plasticity and stress response transmission [59].

### 9.2. Choline

Among the foods that are high in cholesterol per gram are eggs, beef liver, chicken liver, and meats. Choline has a variety of functions in the human body, ranging from cell structure to neurotransmitter production. Its deficiency, on the other hand, has an impact on a variety of ailments, including liver disease, atherosclerosis, and neurological disorders. Neuroplasticity in the adult central nervous system can be altered by replenishing with B vitamins like folic acid and riboflavin as well as choline [135, 136]. On days 0 and 14, Balb/c mice were sensitized with 100 g ovalbumin and then challenged with aerosolized ovalbumin on days 25–27. On days 14–27, mice were given 1 mg kg–1 choline either oral gavage or intranasal method. In addition, mice were given 100 mg kg–1 of lipoic acid as a conventional antioxidant. In bronchoalveolar lavage (BAL) fluid, total cell counts, eosinophils, and eosinophil peroxidase (EPO) activity were measured. In BAL fluid, levels of reactive oxygen species (ROS), lipid peroxidation, and isoprostanes were assessed. The levels of IL-13 and tumor necrosis factor-alpha (TNF-) in BAL fluid and spleen cell culture supernatant were also evaluated. After the final ovalbumin challenge, the expression of the nuclear factor B (NFB) p65 protein was examined in lungs' nuclear and cytosolic extracts. Treatment with choline and lipoic acid significantly reduced eosinophilic infiltration and EPO activity in BAL fluid compared to mice that were exposed to ovalbumin. Reducing the levels of ROS and isoprostanes in BAL fluid was achieved through the use of choline and lipoic acid therapy [137, 138].

## 10. Novel Therapeutic Agent

### 10.1. Phytocannabin

There has recently been a surge in interest in researching cannabis-based products for medical purposes [139]. However, research in the field of neurology is still lacking, and further randomized double-blind placebo studies are needed [140]. People who have seizures linked to certain epileptic syndromes and MS patients who experience spasms have been shown to benefit from Class I evidence of the goods being tested for suitability keeping in mind that not all cannabis-based products are created equal is essential in the process of labeling [141]. In the absence of FDA oversight, products sold and marketed for human consumption are neither regulated nor subjected to rigorous testing. Furthermore, there is still debate on brain inhibition. Other potential symptom exacerbating characteristics of various marijuana compounds Marijuana's chemical constituents interact with other drugs. There is still more to learn [139]. Obtaining information regarding the use of these products is common, not by medical providers, but by other patients, advertisements, or media sources [142].

MS symptoms can be relieved with a combination of 9-THC and CBD, which led to the development of the first legal PCB medication, Sativex. As a neuroprotective agent, CBD's ability to influence immune cell activity in the central nervous system (CNS) and limit oxidative stress is very promising. In particular, CBD's ability to modulate CNS immune cell activity and limit oxidative stress is very promising. However, it is important to note that previous antioxidant and anti-inflammatory-based treatments for neurodegenerative diseases have had minimal clinical efficacy in many cases [143]. There is currently very little human data on PCB effects in neurodegenerative illnesses aside from the favorable evidence on the benefits of SCEs on MS symptoms gathered over the last decade. For patients with Alzheimer's disease, Parkinson's disease, and other neurodegenerative diseases, clinical trials examining the effects of PCBs on both disease progression and symptom control are needed. It does seem that PCB-based therapies, regardless of the target condition, are well tolerated, which is a positive sign for future trials. Antioxidant properties of other PCBs have been overlooked, but they will be better suited to specific illness. 9-THCV, for example, negatively regulates both neuronal cell death and probably results immunological response in models of Parkinson's disease, while reducing signs of bradykinesia.

### 10.2. Rosmarinus officinalis

Neuroprotective properties have been found in Rosmarinus officinalis, and it is a surprising discovery. Alzheimer's disease and dementia are examples of such disorders. Rosemary is a woman who proved inhibitory activity using the two enzymes such as butyryl cholinesterase (BChE) and acetylcholinesterase (AChE). Acetylcholine-cholinesterase is responsible for its breakdown. These are also responsible for the essential oils of the plant [144]. Enhancing Rosemary has been shown to reduce total choline levels in the brain. Memory loss, anxiety, and depression are all symptoms of Alzheimer's disease [135, 136]. Two more studies show that the ability to protect the brain is beneficial. R. officinalis is a medicinal plant. The first is polyphenols, which are found in rosemary. Stress proteins, which play a role in disease, were found to be inhibited by the extract the neurodegenerative process [145]. Rosemary has also been shown to increase the production of nerve growth factor (NGF), a protein that is essential for nerve growth and maintenance. Alzheimer's disease may benefit from increased NGF levels. So, this study has showed that Rosemary has a lot of roles to expand as a plant's neuroprotective substance.

### 10.3. Nano Therapy

**Figure 3:**
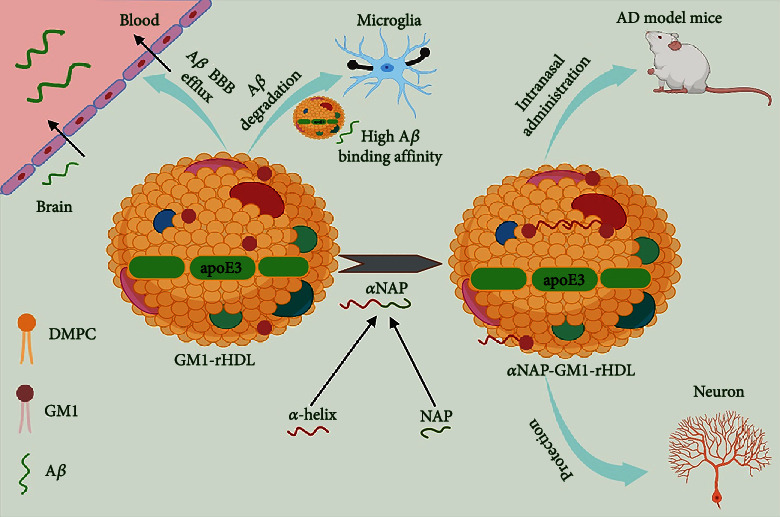
Monosialotetrahexosyl ganglioside-incorporated reconstituted high-density lipoprotein (GM1-rHDL) possesses antibody-like high binding affinity to A*β*, facilitates A*β* degradation by microglia and A*β* efflux across the blood-brain barrier (BBB), and simultaneously allows the efficient loading of neuroprotective agents, serving as a nanoparticulate drug delivery system for the combination therapy of AD [155].

Alzheimer's disease treatment and prevention have used nanotherapeutic methods (Figure 3). Three barriers protect the brain and spinal cord: the blood-brain barrier (BBB), the blood-cerebrospinal fluid barrier (BCSFB), and the ependymal barrier (CNS). As the cerebrospinal fluid (CSF) flows through into the choroid plexus of the ventricles, the BCSFB acts as a barrier between the fluid and brain tissue. Communication between the central nervous and blood is managed by the biological membrane (BBB) [146]. Aside from protecting the CNS from harmful chemicals and promoting hemostasis, the BBB also blocks the delivery of medications to the CNS. The BBB [147] allows only extremely lipid-soluble molecules with a molecular weight (MW) of less than 400 Da to pass through. The BBB is changed in AD, according to various findings.

BBB disruption could be both a cause and a symptom of Alzheimer's disease. Three types of BBB damage have been identified as contributing to the start of AD: leaking of unwanted substances from the bloodstream into the CNS, transport system malfunction, and changes in protein expression in endothelial cells [146]. Medication delivery may be managed to improve by trying to disrupt the BBB, which increases BBB permeability, reduces efflux transporter expression, and reduces CSF reabsorption. Due to a decreased BBB in some cases of Alzheimer's disease, medication distribution to the CNS is diminished, which is extremely damaging to drug delivery [146].

Because such nanoparticles have a high surface to volume ratio and can be synthesized and characterized with desired ligands, nanotherapeutic methods that would be used in noninvasive frameworks have shown promise in overcoming the BBB impediment. Multiple nanotherapeutic methods, such as targeting A, cholinesterase downregulation, and dissolving fibrinogen clots, outperformed conventional therapy significantly [148]. Genetically restricted or regulated expression of the A peptide, suppression of the fibrillation process, and removing accumulated A amyloids from the brain have all been utilized to target A plaques. The amyloid precursor protein (APP) is cleaved at the N-terminus by BACE1's protease activity, resulting in the A-amyloid peptide. The BACE1-siRNA was efficiently scattered using synthesis of nanomethods and was using naturally inert exosomes to limit its activity. To reduce immunogenicity and maximize the efficacy of silencing, the targeted exosome nanocarriers were obtained from C57BL/6 mice's bone marrow cells. Another way to counter A plaques is to slow or stop the nucleation-dependent process that produces fibrils and the resulting plaques. DSPC-Chol liposome NPs functionalized with an A monoclonal antibody were checked on postmortem AD brains (A-MAb) [149] that backed up the effectiveness of the proposed technique. The removal of brain plaques caused by decrepitude is the third procedure. Gold (Au) NPs and reconstituted high-density lipoprotein NPS expanded BBB permeability and, as a result, targeted efficacy in resolving and destroying aggregates. It was highly suggested by innumerable epidemiological studies, which was eventually approved by the FDA, following the failures of many clinical trials connected to the A cascade concept. Using modified poly (n-butyl cyanoacrylate) NPs with polysorbate 80 and additionally chitosan NPs, nanotechnology-based treatments deliver the cholinesterase inhibitor rivastigmine to the brains of Wistar rats via intravenous and intranasal injection, respectively [150, 151]. Cerebrovascular risk factors have been linked to the severity of Alzheimer's disease and cognitive impairment in some epidemiological studies. When cerebrovascular dysfunction occurs alongside Alzheimer's disease, the pathological symptoms are increased [152]. Fibrinogen can enter AD brains through disrupted BBBs, resulting in abnormal clots, especially when A is present. Based on this information, EMT zeolite NPs were developed to inhibit A interaction with fibrinogen and the rate of A fibrillation. Like uncovered EMT zeolite NPs, their fibrinogen binding strength was inhibited after they were covered by the corona layer [153]. Safety and toxicological methods have limited translational pathways even though clinical research based on nanotechnology is still active, as safety concerns about nanoparticle-mediated adverse effects have become more significant when medication delivery to the central nervous [154]. It is becoming easier for pharmaceutical companies to conduct preclinical evaluations and high-throughput screenings of small compounds because of the development of brain-mimetic 3D-culture models.

## 11. Neuroprotection: Challenges and Opportunities

**Figure 4:**
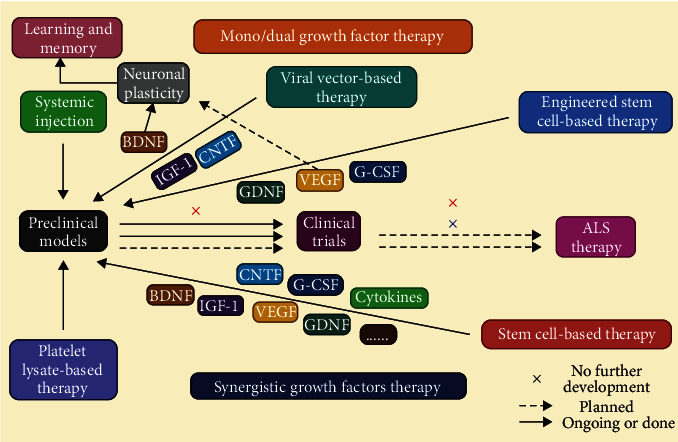
Individual neurotrophic growth factor therapy for synergistic effects. (i) Synergistic growth factor therapy. (ii) Mono/dual growth factor therapy. (iii) ALS therapy [177].

Demonstrating the effectiveness of any neuroprotective therapy in people is the main challenge in neuroprotection. Classical pharmacology, complicated approaches such as deep brain stimulation, and traditional pharmaceutical equipment are all being explored as part of neuroprotective therapy (ENT) [156]. Recent developments in both experimental technique and the design of clinical trials have prompted cautious hope. Such concerns as therapeutic window, dose-response profile, and CNS penetration have been more consistently addressed in experimental pharmacological investigations [157]. There are numerous methods and assays that may be used to determine the status of the OxS biomarkers in a range of biological samples, which is a major advantage but also a downside. Each method has its own set of pros and limitations, as well as practicality and economic considerations [158]. The interpretation of data may also be complicated by a number of pre- and post-analytical difficulties, which require further standardization such as preanalytical issues, analytical issues and, postanalytical issues [159]. However, in diseases such as Parkinson's (PD) and Alzheimer's (AD), neuroprotective interventions are required to reduce neuronal death, whereas in conditions such as ALS (Figure 4), autism spectrum disorders (ASD), spinal cord injuries, and other such conditions, restoration or both protection and restoration are required to restore neurons [156]. A lethal form of persistent neurodegeneration, ALS (amyotrophic lateral sclerosis) is characterized by the presence of proteinaceous, ubiquitinated cytoplasmic inclusions in affected motor neurons as well as in cells surrounding these neurons [160]. Apoptosis may have a role in neurodegeneration, according to some research, but others disagree. This controversy must be settled since it is therapeutically significant. Clarifying MN death pathways gives reasonable targets for future ALS treatments. A primary focus of study needs to be on finding models of motor neuron degeneration that are the most accurate representations of motor neuron death in clinical ALS (outstanding issues). Identification of MN cell death pathways and evaluation of medications and biological substances for neuroprotective properties are both made possible via the use of animal and cell model system research [161]. Traumatic brain injury (TBI) is the leading cause of mortality and disability in those under 45 worldwide. The knowledge of pathophysiological events has been expanded as a result of numerous experimental and clinical studies of biomechanical injury and tissue damage. This information has the potential to serve as the foundation for the development of novel treatment techniques as well as the enhancement of treatment strategies that are already in use [162]. Dementia treatment differs based on the kind of dementia detected in people who have had traumatic brain injuries. When treating Alzheimer's disease or any form of dementia, patients with and without a previous history of traumatic brain injury should adhere to the same treatment protocols [163]. In the case of mild traumatic brain injury (TBI), neuropsychological tests are often used in combination with imaging to assess brain function. Memory, focus, information processing, executive functioning, and response speed are some of the cognitive abilities that may be assessed with these tests [164].

Neuroprotection is a treatment method that modifies the consequences of the ischemia cascade or facilitates reperfusion in order to protect neurons from suffering irreparable damage. This is done in the hopes of preventing neuronal death [165]. Despite the fact that various medicines have shown neuroprotective effects in preclinical studies, the translation of those findings to clinical trials has failed to show any significant impact. The NDDs may be distinguished from one another in large part by the anatomical locations that exhibit neuronal dysfunction, biochemical and structural changes in protein indicators, and neuronal cell diseases including the deposition of protein(s), as well as alterations in genetics and epigenetics [166]. Since it is undeniable that OxS plays a role in the etiology of many chronic degenerative illnesses as well as the aging process, several investigations into the potential advantages of antioxidant treatment have been made. Exogenous supplementation is justified in order to preserve the general public's wellness and health by avoiding the onset of illness, its progression, and its repercussions. Genome instability is mostly caused by endogenous stress. It is true that the main force for genomic evolution, genetic diversity, is necessary for physiological activities. The goal of the most recent diagnostic studies is to create readily identifiable biomarkers from saliva or blood to discriminate between the many types of neurological diseases.

### 11.1. Neuro-Imaging in the Identification of Severe Disorders Caused by Neurodegeneration

The unique protein that aggregates characterizes each neurodegenerative disease type. Extensive research has recently been conducted on disease-modifying medicines for neurodegenerative diseases (e.g., Alzheimer's disease and tauopathies), with the hope of developing them in the near future. Urgently needed for the correct diagnosis of neurodegenerative diseases as well as the facilitation of the creation of disease-modifying therapies are disease-specific biomarkers that are both straightforward and applicable [167]. Characteristic imaging findings in neurodegenerative diseases are described in several diagnostic criteria. Furthermore, only a small number of current diagnostic criteria for neurodegenerative disorders have identified neuroimaging methods as biomarkers that might gauge the pathological changes taking place in neurodegenerative disease patients' brains. The importance of neuroimaging methods as biomarkers are discussed here for various neurodegenerative disorders such as magnetic resonance imaging, single photon emission computed tomography (SPeCT) and positron emission tomography (PeT), dopamine transporter imaging, 123iodine-metaiodobenzylguanidine, myocardial scintigraphy, A*β* imaging, and Tau imaging. Functional neuroimaging methods are used to diagnose the most common CNS illnesses (Parkinson's disease (PD), Alzheimer's disease (AD), Huntington's disease (HD), amyotrophic lateral sclerosis (ALS), and Multiple sclerosis (MA)) [168].

### 11.2. Anatomical Identifications of Neuronal Losses in relation to Clinical Symptoms

In order to get a proper grasp of the early symptoms, identification of the anatomical locations is required. For instance, the regions of the brain known as the entorhinal cortex, neocortex, hippocampus, and limbic system are responsible for symptoms such as cognitive decline, dementia, and other alterations in high-order brain functions, whereas thalamus, the basal ganglia, motor cortical, and brain stem areas are primarily responsible for disturbances in body movements. The majority of the time and combinations of these different kinds of symptoms are found throughout the evolution of illnesses that follow region-specific neurodegenerations.

The computational models that are going to be given here perform certain alterations on three-dimensional (3D) ensembles of neurons that represent healthy persons. The data from digital photographs of brain tissue were utilized to construct a reasonable three-dimensional arrangement of neurons with statistical features that were compatible with experimental data in order to establish these first ensembles [169]. This computational technology was developed in the past. To be more specific, the prior technique measured the microcolumnarity of a particular tissue slice in order to generate, with the help of a density map computation, the 3D representation of neurons that had the same statistical features as those seen in the experimental tissue [170]. After they have been created, these preliminary ensembles will be used as the foundation for stochastic simulations that will investigate different models of deletion and displacement. These simulations will begin after the preliminary ensembles have been formed. The models that are going to be described here consider groups of neurons that are arranged in a Cartesian coordinate system. This is done in order to facilitate the computations being performed with more ease [171]. We find that this limitation has no discernible impact on the scope of either our work or our conclusions when we contrast the results from our models with data obtained from straight sections of tissue that were locally fitted to this coordinate system. This method is widely used and has been implemented by others in order to keep from becoming confused by the impacts of curved areas. However, due to the nature of the methodology that has been presented in this article, it is possible to make straightforward generalizations to other coordinate systems. These other coordinate systems might be able to better describe curved regions of the brain, such as those that can be found in the lip or fundus of any sulcus [172].

### 11.3. Brain Mapping: A Diagnostic Tool for Neurodegenerative Diseases

The scientific community has been captivated for a very long time by the prospect of producing maps that may localize cognitive processes and alterations caused by illness to specific areas of the brain. Brodmann's cytoarchitectonic map, which was created in the early twentieth century and separated 52 cortical subregions based on their distinctions in thickness, lamination, neuronal type, and staining characteristics, is perhaps the most lauded brain mapping feat to date. Since the development of noninvasive neuroimaging techniques, brain maps have progressed to the point that they are now digital atlases that are highly complex, multidimensional, and multimodal. These atlases cover the whole of a human's life and depict the course of a number of disorders [173]. Since the middle of the 1990s, many potent brain mapping methods have been developed. A lot of people rely on computational anatomy, a mathematical approach to modeling the brain in which brain surfaces and subvolumes are viewed as complex geometrical patterns and are modeled as 3D continuous mesh models or deformable shapes that can be averaged and combined across subjects, and on which statistics can be defined [174]. Thus, the anatomical pictures are changeable templates that may be elastically or fluidly reshaped into a comparable shape, most often onto the average of the research group, an atlas average, or the brain shape of another individual [175]. Some of these methods employ surface markers as constraints to explicitly represent the anatomy of the brain (e.g., sulci). Without losing the underlying subtleties in the measure of interest, these strategies easily enable the correct alignment of surface-specific geometrical patterns (such as gyri) and assist in accurately colocalizing identical cortical and subcortical areas (e.g., cortical thickness, functional activation, or gray matter density). The ability to describe cortical and subcortical illness patterns and identify subtle disease-associated alterations is enhanced by the ensuing anatomical coregistration [176].

## 12. Conclusion and Future Perspective

Many neurodegenerative diseases, such as Alzheimer's, Parkinson's, and amyotrophic lateral sclerosis, are caused by free radical damage to human nerve cells. This gang of neurodegenerative diseases is to start blaming for the obliteration of DNA, lipids, and proteins. Phytochemicals, magnesium, choline, and turmeric in our diet all play a role in neuroplasticity by acting as neuroprotective agents. This review illustrates the different neuroprotective agents and their functions.

Neuroprotective agents have been shown to be beneficial in a variety of models, including rats, mice, and drosophila, as well as humans. Various neurodegenerative infections can be treated with bioactive molecules found in medicinal plants because medicinal plants are shown to have neuroprotective properties. Alzheimer's disease people can benefit from AGE, a supplement food that improves cognitive function, as a supplement.

Dopaminergic neurons in Parkinson's disease are protected by Bacoside A and Donezil, and allium stadium has been shown to protect cells from oxidative stress while also inhibiting A aggregation and fibril formation. Clinical trials have shown neuroprotective effects, but this is not sufficient. A large sample size is needed for the clinical trial. This review could be helpful for future research priorities on various phytochemicals that can be used as a medicine because they had been shown already a wide range of health benefits such as inhibiting of A*β* aggregation and formation of fibrils, decreasing the amount of A*β* fibris, and protecting the dopaminergic neurons in Parkinson's disease and also are capable of protecting cells from oxidative stress. Despite numerous clinical studies demonstrating neuroprotective financial advantages, more research needs to be done. Large numbers of people should be involved in the research. There are numerous phytochemicals that can be used in medicine, and a review of this material could help guide future research because a wide range of health benefits had already been illustrated.

## Figures and Tables

**Table 1:** Neuroprotective agent in intensive care for hemorrhagic stroke, acute ischemic stroke, and brain trauma management.

Neuroprotective agents	Class	Therapeutic applications	Recommended dosage	Witness the people	Result	References
Polyarginine R18 NA-1 (TAT-NR2B9c)	Glutamate blockers	Ischemic stroke	1000 nmol/kg	Rat	Maintained functional outcomes as well as reduced infarct volume.	[67–71]
Magnesium sulfate	Glutamate blockers/NMDA channels blocker	Hemorrhagic and ischemic stroke, traumatic brain injuries	Up to 65 mmol/day	Patients were indeed human beings.	One explanation Mgso4 had such a successful aspect was the fact that it reduced late-onset ischemia of the central nervous system (DCI)	[60, 72–78]
Rosuvastatin and simvastatin atorvastatin, mevastatin	Statins	Ischemic stroke	Up to 20 mg/kg/day	Mice and humans are the subjects of this investigation	a decrease in mortality, a decrease in the size of something like the infarct, and an increase in cerebral blood flow	[61–63, 65]
Melatonin	Hormone	Hemorrhagic and ischemic stroke, traumatic brain injuries	Up to 200 mg/kg/day	Mice and rats from New Zealand's white rabbit electorate	Reduce oxidative damage by attempting to prevent endothelial cell vasospasm and apoptosis	[66, 79]
Erythropoietin	Growth factor for determining the ability	Stroke, both hemorrhagic and ischemic, and traumatic injury toward the brain	Up to 5000 units/kg	Rabbit, rat, mice	a smaller infarct, less vasospasm, and an effective therapy in terms of function	[80, 81]
Cyclosporin A (CsA) and FK506 (tacrolimus)	Immunosuppressant	Strokes, brain trauma, ischemic stroke	As much as 10 milligrams per kilogram for CsA FK506 has a full dose of 6 milligrams per kilogram	Rat	Improved functional recovery, significantly reduced volume of infarct tissue	[82, 83]

**Table 2:** Neuropsychopharmacological consequences of medicinal plants.

Bioactive compounds	Medicinal plants	Therapeutic applications	Impact/action	References
Carvone, allyl tetrasulfide diallyl disulfide, and diallyl trisulfide	*Allium sativum*	Neuroprotection	Simple recollection, gliosis, and oxidative stress are all directly affected	[110]
Bacoside	*Bacopa monnieri*	Protective lead for Alzheimer's chronic conditions	Defend the brain from oxidative damage and the decreased cognitive function that comes with increased age	[111]
Asiatic acid, M-adeacamic acid, and brahmaside as well as flavonoids madecassoside and madesiatic acid	*Centella asiatica*	Antimicrobial, anti-inflammatory, anticancer, neuroprotective, cytotoxic	Preventing the emergence of amyloid plaque in Alzheimer's disease, as well as reducing dopamine neurotoxicity in Parkinson's disease, is the highest priorities of enzyme inhibition	[112]
Monoterpenes (linalool, alpha-terpinyl acetate, andnerol acetate) sesquiterpene esters (Suchar malhangunoil, Balkan gun in, valerenal, global) Vioridiflorol, cubenol, agarouran derivatives, diterpenoids such as lupeol, pristine in, pristine in, zyeylosteral, alkaloids such as celapenin, celapenigin, panculatine, celestine maymyrone, tatty acids, steroids such as serpentine, flavonoids, benzoic acid, and vitamin C	*Celastrus paniculatus*	An expected performance for neuroprotection in the management of neurodegenerative diseases the same as Alzheimer's and other neuronal disorders	Inhibits the levels of noradrenaline, dopamine, and 5-hydroxy tryptamine	[113]
Sesquiterpene alkaloid	*Huperzia Serrata*		Neuroprotective against a beta-amyloid peptide fragment, potent AChE inhibitor	[114]
6-gingerol	*Zingiber Officinale*	Treatment of Alzheimer's disease		[115]
Clerodane diterpenes	*Croton yanhuii*	Treatment of Alzheimer's disease		[116]
Xylocarpin B, Xylocarpin G	*Xylocarpus granatum*	Neuroprotective property		[117]
Resveratrol	*Vitis vinifera*	Neuritogenesis, neuroinflammation, neuroprotection property		[118]

## Data Availability

All data are available within the text.

## References

[1] Sato H., Shibata M., Shimizu T. (2013). Differential cellular localization of antioxidant enzymes in the trigeminal ganglion. *Neuroscience*.

[2] Navarro-Yepes J., Zavala-Flores L., Anandhan A. (2014). Antioxidant gene therapy against neuronal cell death. *Pharmacology & Therapeutics*.

[3] Phaniendra A., Jestadi D. B., Periyasamy L. (2015). Free radicals: properties, sources, targets, and their implication in various diseases. *Indian Journal of Clinical Biochemistry*.

[4] Valko M., Leibfritz D., Moncol J., Cronin M. T. D., Mazur M., Telser J. (2007). Free radicals and antioxidants in normal physiological functions and human disease. *The International Journal of Biochemistry & Cell Biology*.

[5] Nordberg J., Arnér E. S. J. (2001). Reactive oxygen species, antioxidants, and the mammalian thioredoxin system^1^. *Free Radical Biology & Medicine*.

[6] Rahman M. M., Islam M. R., Shohag S. (2022). The multifunctional role of herbal products in the management of diabetes and obesity: a comprehensive review. *Molecules*.

[7] Rauf A., Badoni H., Abu-Izneid T. (2022). Neuroinflammatory markers: key indicators in the pathology of neurodegenerative diseases. *Molecules*.

[8] Rahman M. M., Islam M. R., Shohag S. (2022). Multifaceted role of natural sources for COVID-19 pandemic as marine drugs. *Environmental Science and Pollution Research*.

[9] Rahman M. M., Islam F., Parvez A. (2022). *Citrus limon* L. (lemon) seed extract shows neuro-modulatory activity in an *in vivo* thiopental-sodium sleep model by reducing the sleep onset and enhancing the sleep duration. *Journal of Integrative Neuroscience*.

[10] Simonian N. A., Coyle J. T. (1996). Oxidative stress in neurodegenerative diseases. *Annual Review of Pharmacology and Toxicology*.

[11] Rahman M. M., Ferdous K. S., Ahmed M. (2020). Emerging promise of nanoparticle-based treatment for Parkinson’s disease. *Biointerface Research in Applied Chemistry*.

[12] Rahman M. M., Islam M. R., Islam M. T. (2022). Stem cell transplantation therapy and neurological disorders: current status and future perspectives. *Biology*.

[13] Finkbeiner S. (2011). Huntington’s disease. *Cold Spring Harbor Perspectives in Biology*.

[14] Berman T., Bayati A. (2018). What are neurodegenerative diseases and how do they affect the brain?. *Frontiers for Young Minds*.

[15] Venkatesan R., Ji E., Kim S. Y. (2015). Phytochemicals that regulate neurodegenerative disease by targeting neurotrophins: a comprehensive review. *BioMed Research International*.

[16] Dugger B. N., Dickson D. W. (2017). Pathology of neurodegenerative diseases. *Cold Spring Harbor Perspectives in Biology*.

[17] Roberts J. S., Patterson A. K., Uhlmann W. R. (2020). Genetic testing for neurodegenerative diseases: ethical and health communication challenges. *Neurobiology of Disease*.

[18] Kocot J., Luchowska-Kocot D., Kiełczykowska M., Musik I., Kurzepa J. (2017). Does vitamin c influence neurodegenerative diseases and psychiatric disorders?. *Nutrients*.

[19] Sawicka-Glazer E., Czuczwar S. J. (2014). Vitamin C: a new auxiliary treatment of epilepsy?. *Pharmacological Reports*.

[20] Warner T. A., Kang J. Q., Kennard J. A., Harrison F. E. (2015). Low brain ascorbic acid increases susceptibility to seizures in mouse models of decreased brain ascorbic acid transport and Alzheimer's disease. *Epilepsy Research*.

[21] Schulte P. A., Bumett C. A., Boeniger M., Johnson J. (1996). Neurodegenerative diseases: occupational occurrence and potential risk factors, 1982 through 1991. *American Journal of Public Health*.

[22] Frenkel K., Chrzan K., Troll W., Teebor G. W., Steinberg J. J. (1986). Radiation-like modification of bases in DNA exposed to tumor promoter-activated polymorphonuclear leukocytes. *Cancer Research*.

[23] Amer J., Ghoti H., Rachmilewitz E., Koren A., Levin C., Fibach E. (2006). Red blood cells, platelets and polymorphonuclear neutrophils of patients with sickle cell disease exhibit oxidative stress that can be ameliorated by antioxidants. *British Journal of Haematology*.

[24] Joseph N., Zhang-James Y., Perl A., Faraone S. V. (2015). Oxidative stress and ADHD: a meta-analysis. *Journal of Attention Disorders*.

[25] Agrawal M., Biswas A. (2015). Molecular diagnostics of neurodegenerative disorders. *Frontiers in Molecular Biosciences*.

[26] Burton G. J., Jauniaux E. (2011). Oxidative stress. *Best Practice & Research. Clinical Obstetrics & Gynaecology*.

[27] Hayashi M. (2009). Oxidative stress in developmental brain disorders. *Neuropathology*.

[28] Morales-Gonzalez J. A., Morales-Gonzalez A., Madrigal-Santillan E. O. (2016). *A master regulator of oxidative stress-the transcription factor Nrf2*.

[29] Coskun P. E., Beal M. F., Wallace D. C. (2004). Alzheimer’s brains harbor somatic mtDNA control-region mutations that suppress mitochondrial transcription and replication. *Proceedings of the National Academy of Sciences of the United States of America*.

[30] Mecocci P., MacGarvey U., Beal M. F. (1994). Oxidative damage to mitochondrial DNA is increased in Alzheimer's disease. *Annals of Neurology*.

[31] Reeve A. K., Krishnan K. J., Turnbull D. (2008). Mitochondrial DNA mutations in disease, aging, and neurodegeneration. *Annals of the New York Academy of Sciences*.

[32] Gibson G. E., Zhang H., Sheu K. F. R. (1998). A *α*-ketoglutarate dehydrogenase in alzheimer brains bearing the APP670/671 mutation. *Annals of Neurology*.

[33] Cardoso S. M., Proença M. T., Santos S., Santana I., Oliveira C. R. (2004). Cytochrome *c* oxidase is decreased in Alzheimer 's disease platelets. *Neurobiology of Aging*.

[34] Tang S. W., Helmeste D. M., Leonard B. E. (2017). Neurodegeneration, neuroregeneration, and neuroprotection in psychiatric disorders. *Modern Trends in Psychiatry*.

[35] Bobkova N. V., Poltavtseva R. A., Leonov S. V., Sukhikh G. T. (2020). Neuroregeneration: regulation in neurodegenerative diseases and aging. *The Biochemist*.

[36] Nagappan P. G., Chen H., Wang D. Y. (2020). Neuroregeneration and plasticity: a review of the physiological mechanisms for achieving functional recovery postinjury. *Military Medical Research*.

[37] Saikia D. (2017). Potential drug targets for neuroregeneration and repair. *Journal of Analytical & Pharmaceutical Research*.

[38] Toricelli M., Pereira A., Souza Abrao G. (2021). Mechanisms of neuroplasticity and brain degeneration: strategies for protection during the aging process. *Neural Regeneration Research*.

[39] Dorszewska J., Kozubski W., Waleszczyk W., Zabel M., Ong K. (2020). Neuroplasticity in the pathology of neurodegenerative diseases. *Neural Plasticity*.

[40] Demarin V., Morović S., Béné R. (2014). Demarin. *Periodicum Biologorum*.

[41] Chang Y. (2014). Reorganization and plastic changes of the human brain associated with skill learning and expertise. *Frontiers in Human Neuroscience*.

[42] Mateos-Aparicio P., Rodríguez-Moreno A. (2019). The impact of studying brain plasticity. *Frontiers in Cellular Neuroscience*.

[43] Stephenson R. (1993). A review of neuroplasticity: some implications for physiotherapy in the treatment of lesions of the brain. *Physiotherapy*.

[44] Sharma R., Kabra A., Rao M. M., Prajapati P. K. (2018). Herbal and holistic solutions for neurodegenerative and depressive disorders: leads from Ayurveda. *Current Pharmaceutical Design*.

[45] Newman C. J., O’Regan M., Hensey O. (2006). Sleep disorders in children with cerebral palsy. *Developmental Medicine and Child Neurology*.

[46] Maier M., Ballester B. R., Verschure P. F. M. J. (2019). Principles of neurorehabilitation after stroke based on motor learning and brain plasticity mechanisms. *Frontiers in Systems Neuroscience*.

[47] Johansen-Berg H., Dawes H., Guy C., Smith S. M., Wade D. T., Matthews P. M. (2002). Correlation between motor improvements and altered fMRI activity after rehabilitative therapy. *Brain*.

[48] Benz S., Sellaro R., Hommel B., Colzato L. S. (2016). Music makes the world go round: the impact of musical training on non-musical cognitive functions-a review. *Frontiers in Psychology*.

[49] Rahman M. M., Mim S. A., Islam M. R. (2022). Exploring the recent trends in management of dementia and frailty: focus on diagnosis and treatment. *Current Medicinal Chemistry*.

[50] Seidl S. E., Potashkin J. A. (2011). The promise of neuroprotective agents in Parkinson?s disease. *Frontiers in Neuroscience*.

[51] Turrin N. P., Rivest S. (2006). Molecular and cellular immune mediators of neuroprotection. *Molecular Neurobiology*.

[52] Reichardt L. F. (2006). Neurotrophin-regulated signalling pathways. *Philosophical Transactions of the Royal Society, B: Biological Sciences*.

[53] Huang E. J., Reichardt L. F. (2001). Neurotrophins: roles in neuronal development and function. *Annual Review of Neuroscience*.

[54] Aloe L., Rocco M. L., Bianchi P., Manni L. (2012). Nerve growth factor: from the early discoveries to the potential clinical use. *Journal of Translational Medicine*.

[55] Petruska J. C., Mendell L. M. (2009). Nerve growth factor. *Encyclopedia of Neuroscience*.

[56] Kalani A., Tyagi A., Tyagi N. (2014). Exosomes: mediators of neurodegeneration, neuroprotection and therapeutics. *Molecular Neurobiology*.

[57] Liu W., Bai X., Zhang A., Huang J., Xu S., Zhang J. (2019). Role of exosomes in central nervous system diseases. *Frontiers in Molecular Neuroscience*.

[58] Jancic D., Lopez De Armentia M., Valor L. M., Olivares R., Barco A. (2009). Inhibition of cAMP response element-binding protein reduces neuronal excitability and plasticity, and triggers neurodegeneration. *Cerebral Cortex*.

[59] Ichiki T. (2006). Role of cAMP response element binding protein in cardiovascular remodeling. *Arteriosclerosis, Thrombosis, and Vascular Biology*.

[60] Panahi Y., Mojtahedzadeh M., Najafi A., Rajaee S. M., Torkaman M., Sahebkar A. (2018). Neuroprotective agents in the intensive care unit. *Journal of Pharmacopuncture*.

[61] Jain M. K., Ridker P. M. (2005). Anti-inflammatory effects of statins: clinical evidence and basic mechanisms. *Nature Reviews. Drug Discovery*.

[62] Parizadeh S. M. R., Azarpazhooh M. R., Moohebati M. (2011). Simvastatin therapy reduces prooxidant-antioxidant balance: results of a placebo-controlled cross-over trial. *Lipids*.

[63] Laufs U., Gertz K., Huang P. (2000). Atorvastatin upregulates type III nitric oxide synthase in thrombocytes, decreases platelet activation, and protects from cerebral ischemia in normocholesterolemic mice. *Stroke*.

[64] Sharma R., Prajapati P. K. (2020). Predictive, preventive and personalized medicine: leads from ayurvedic concept of Prakriti (human constitution). *Current Pharmacology Reports*.

[65] Moon G. J., Kim S. J., Cho Y. H., Ryoo S., Bangc O. Y. (2014). Antioxidant effects of statins in patients with atherosclerotic cerebrovascular disease. *Journal of Clinical Neurology*.

[66] Koh P. O. (2008). Melatonin regulates nitric oxide synthase expression in ischemic brain injury. *The Journal of Veterinary Medical Science*.

[67] Milani D., Cross J. L., Anderton R. S., Blacker D. J., Knuckey N. W., Meloni B. P. (2017). Neuroprotective efficacy of poly-arginine R18 and NA-1 (TAT-NR2B9c) peptides following transient middle cerebral artery occlusion in the rat. *Neuroscience Research*.

[68] Milani D., Knuckey N. W., Anderton R. S., Cross J. L., Meloni B. P. (2016). The R18 Polyarginine peptide is more effective than the TAT-NR2B9c (NA-1) peptide when administered 60 minutes after permanent middle cerebral artery occlusion in the rat. *Stroke Research and Treatment*.

[69] Rahman M. M., Rahaman M. S., Islam M. R. (2022). Role of phenolic compounds in human disease: current knowledge and future prospects. *Molecules*.

[70] Rahman M. M., Islam F., Afsana Mim S. (2022). Multifunctional therapeutic approach of nanomedicines against inflammation in cancer and aging. *Journal of Nanomaterials*.

[71] Rahman M. M., Islam M. R., Shohag S. (2022). Microbiome in cancer: role in carcinogenesis and impact in therapeutic strategies. *Biomedicine & Pharmacotherapy*.

[72] Afshari D., Moradian N., Rezaei M. (2013). Evaluation of the intravenous magnesium sulfate effect in clinical improvement of patients with acute ischemic stroke. *Clinical Neurology and Neurosurgery*.

[73] Mizukoshi G., Katsura K. I., Katayama Y. (2005). Urinary 8-hydroxy-2′-deoxyguanosine and serum S100*β*in acute cardioembolic stroke patients. *Neurological Research*.

[74] Saver J. L., Starkman S., Eckstein M. (2015). Prehospital use of magnesium sulfate as neuroprotection in acute stroke. *The New England Journal of Medicine*.

[75] Jaakkola J., Mustonen P., Kiviniemi T. (2016). Stroke as the first manifestation of atrial fibrillation. *PLoS One*.

[76] Levi M., Brimble M. (2012). A review of neuroprotective agents. *Current Medicinal Chemistry*.

[77] Van Den Bergh W. M. (2005). Magnesium sulfate in aneurysmal subarachnoid hemorrhage. *Stroke*.

[78] Wong G. K. C., Chan D. Y. C., Siu D. Y. W. (2015). High-dose simvastatin for aneurysmal subarachnoid Hemorrhage. *Stroke*.

[79] Ozdemir D., Tugyan K., Uysal N. (2005). Protective effect of melatonin against head trauma-induced hippocampal damage and spatial memory deficits in immature rats. *Neuroscience Letters*.

[80] Taoufik E., Petit E., Divoux D. (2008). TNF receptor I sensitizes neurons to erythropoietin-and VEGF-mediated neuroprotection after ischemic and excitotoxic injury. *Proceedings of the National Academy of Sciences of the United States of America*.

[81] Grasso G., Tomasello G., Noto M., Alafaci C., Cappello F. (2015). Erythropoietin for the treatment of subarachnoid hemorrhage: a feasible ingredient for a successful medical recipe. *Molecular Medicine*.

[82] Chwiej J., Janeczko K., Marciszko M., Czyzycki M., Rickers K., Setkowicz Z. (2010). Neuroprotective action of FK-506 (tacrolimus) after seizures induced with pilocarpine: quantitative and topographic elemental analysis of brain tissue. *Journal of Biological Inorganic Chemistry*.

[83] Shamsaei N., Khaksari M., Erfani S., Rajabi H., Aboutaleb N. (2015). Exercise preconditioning exhibits neuroprotective effects on hippocampal CA1 neuronal damage after cerebral ischemia. *Neural Regeneration Research*.

[84] Biccard B. M., Sear J. W., Foëx P. (2005). Statin therapy: a potentially useful peri-operative intervention in patients with cardiovascular disease. *Anaesthesia*.

[85] Sever P. S., Poulter N. R., Dahlöf B., Wedel H., Anglo-Scandinavian Cardiac Outcomes Trial Investigators (2005). Different Time Course for Prevention of Coronary and Stroke Events by Atorvastatin in the Anglo-Scandinavian Cardiac Outcomes Trial-Lipid-Lowering Arm (ASCOT-LLA). *The American Journal of Cardiology*.

[86] Elrod J. W., Lefer D. J. (2005). The effects of statins on endothelium, inflammation and cardioprotection. *Drug News & Perspectives*.

[87] Malfitano A. M., Marasco G., Proto M. C., Laezza C., Gazzerro P., Bifulco M. (2014). Statins in neurological disorders: an overview and update. *Pharmacological Research*.

[88] Bacigaluppi M., Hermann D. M. (2008). New targets of neuroprotection in ischemic stroke. *The Scientific World Journal*.

[89] Harborne J. B. (2011). Indian medicinal plants. A compendium of 500 species. Vol. 1; edited by P. K. Warrier, V. P. K. Nambiar and C. Ramankutty. *The Journal of Pharmacy and Pharmacology*.

[90] Kumar Verma S., Article R., Kumar A. (2011). Therapeutic uses of Withania somnifera (Ashwagandha) with a note on withanolides and its pharmacological actions. *Asian Journal of Pharmaceutical and Clinical Research*.

[91] Bashir S., Gilani A. H., Siddiqui A. A. (2010). *Berberis vulgaris* root bark extract prevents hyperoxaluria induced urolithiasis in rats. *Phytotherapy Research*.

[92] Singh N., Nath R., Lata A., Singh S. P., Kohli R. P., Bhargava K. P. (1982). *Withania somnifera* (ashwagandha), a rejuvenating herbal drug which enhances survival during stress (an adaptogen). *International Journal of Crude Drug Research*.

[93] Prabu P. C., Panchapakesan S., Raj C. D. (2013). Acute and sub-acute oral toxicity assessment of the hydroalcoholic extract of Withania somnifera roots in Wistar rats. *Phytotherapy Research*.

[94] Hussein Y., AlShokair S., Ashry K. (2017). Acute and sub-chronic toxicological potential of Withania Somnifera extract on rats. *AJVS*.

[95] Rauf A., Khan I. A., Alnasser S. M., Shah S. U., Rahman M. (2022). Phytochemical analysis and in vitro and in vivo pharmacological evaluation of parthenium hysterophorus linn. *Evidence-Based Complementary and Alternative Medicine*.

[96] Erkut B., Kaya T., Lehmann-Waffenschmidt M. (2018). A fresh look on financial decision-making from the plasticity perspective. *International Journal of Ethics and Systems*.

[97] Rahman M. M., Islam M. R., Emran T. B. (2022). Clinically important natural products for Alzheimer’s disease. *International Journal of Surgery*.

[98] Rahman M. M., Jahan F. I., Mim S. A. (2019). A brief phytochemical investigation and pharmacological uses of citrus seed–a review. *Pharmacology OnLine*.

[99] Shah N., Nariya A., Pathan A., Patel A., Chettiar S., Jhala D. (2018). Neuroprotection effects of Celastrus paniculatus seed oil against monosodium glutamate in human IMR-32 cells. *Annual Research & Review in Biology*.

[100] Konar A., Gautam A., Thakur M. K. (2015). *Bacopa monniera* (CDRI-08) upregulates the expression of neuronal and glial plasticity markers in the brain of scopolamine induced amnesic mice. *Evidence-based Complementary and Alternative Medicine*.

[101] Jyoti A., Sharma D. (2006). Neuroprotective role of *Bacopa monniera* extract against aluminium-induced oxidative stress in the hippocampus of rat brain. *Neurotoxicology*.

[102] Shalini V. T., Neelakanta S. J., Sriranjini J. S. (2021). Neuroprotection with Bacopa monnieri–a review of experimental evidence. *Molecular Biology Reports*.

[103] Patiño-Morales C. C., Jaime-Cruz R., Sánchez-Gómez C. (2022). Antitumor effects of natural compounds derived from allium Sativum on Neuroblastoma: an overview. *Antioxidants*.

[104] Londhe V. P., Gavasane A. T., Nipate S. S., Bandawane D. D., Chaudhari P. D. (2011). Role of garlic (Allium sativum) in various diseases: an overview. *Journal of Pharmaceutical Research and Opinion*.

[105] Ahmed A., Rauf A., Hemeg H. A. (2022). Green synthesis of gold and silver nanoparticles using Opuntia dillenii aqueous extracts: characterization and their antimicrobial assessment. *Journal of Nanomaterials*.

[106] Rahman M. M., Alam Tumpa M. A., Zehravi M. (2022). An overview of antimicrobial stewardship optimization: the use of antibiotics in humans and animals to prevent resistance. *Antibiotics*.

[107] Rahman M. M., Rahaman M. S., Islam M. R. (2021). Multifunctional therapeutic potential of phytocomplexes and natural extracts for antimicrobial properties. *Antibiotics*.

[108] Farooqui T., Farooqui A. A. (2018). Neuroprotective effects of garlic in model systems of neurodegenerative diseases. *Role of the Mediterranean Diet in the Brain and Neurodegenerative Diseases*.

[109] Nillert N., Pannangrong W., Welbat J. U., Chaijaroonkhanarak W., Sripanidkulchai K., Sripanidkulchai B. (2017). Neuroprotective effects of aged garlic extract on cognitive dysfunction and neuroinflammation induced by *β*-amyloid in rats. *Nutrients*.

[110] Hazzaa S. M., Abdelaziz S. A. M., Eldaim M. A. A., Abdel-Daim M. M., Elgarawany G. E. (2020). Neuroprotective potential of allium sativum against monosodium glutamate-induced excitotoxicity: impact on short-term memory, gliosis, and oxidative stress. *Nutrients*.

[111] Abdul Manap A. S., Vijayabalan S., Madhavan P. (2019). *Bacopa monnieri*, a neuroprotective lead in Alzheimer disease: a review on its properties, mechanisms of action, and preclinical and clinical studies. *Drug Target Insights*.

[112] Prakash V., Jaiswal N., Srivastava M. (2017). A review on medicinal properties of Centella asiatica. *Asian Journal of Pharmaceutical and Clinical Research*.

[113] Arora N., Pandey-Rai S. (2012). Celastrus paniculatus, an endangered Indian medicinal plant with miraculous cognitive and other therapeutic properties: an overview. *International Journal of Pharma and Bio Sciences*.

[114] Wang L. S., Zhou J., Shao X. M., Tang X. C. (2003). Huperzine A attenuates cognitive deficits and brain injury after hypoxia-ischemic brain damage in neonatal rats. *Zhonghua Er Ke Za Zhi*.

[115] Jeon S., Hur J., Jeong H. J., Koo B. S., Pak S. C. (2011). SuHeXiang Wan essential oil alleviates amyloid beta induced memory impairment through inhibition of Tau protein phosphorylation in mice. *The American Journal of Chinese Medicine*.

[116] Sun Y., Wang M., Ren Q. (2014). Two novel clerodane diterpenenes with NGF-potentiating activities from the twigs of *Croton yanhuii*. *Fitoterapia*.

[117] Zhou Z. F., Kurtán T., Mándi A. (2016). Novel and Neuroprotective Tetranortriterpenoids from Chinese Mangrove *Xylocarpus granatum* Koenig. *Scientific Reports*.

[118] Anastácio J. R., Netto C. A., Castro C. C. (2014). Resveratrol treatment has neuroprotective effects and prevents cognitive impairment after chronic cerebral hypoperfusion. *Neurological Research*.

[119] AlJohri R., AlOkail M., Haq S. H. (2019). Neuroprotective role of vitamin D in primary neuronal cortical culture. *eNeurologicalSci*.

[120] Teleanu R. I., Chircov C., Grumezescu A. M., Volceanov A., Teleanu D. M. (2019). Antioxidant therapies for neuroprotection-a review. *Journal of Clinical Medicine*.

[121] Ballaz S. J., Rebec G. V. (2019). Neurobiology of vitamin C: expanding the focus from antioxidant to endogenous neuromodulator. *Pharmacological Research*.

[122] Moretti M., Fraga D. B., Rodrigues A. L. S. (2017). Preventive and therapeutic potential of ascorbic acid in neurodegenerative diseases. *CNS Neuroscience & Therapeutics*.

[123] Rebec G. V., Barton S. J., Ennis M. D. (2002). Dysregulation of ascorbate release in the striatum of behaving mice expressing the Huntington’s disease gene. *The Journal of Neuroscience*.

[124] Lee P., Ulatowski L. M. (2019). Vitamin E: mechanism of transport and regulation in the CNS. *IUBMB Life*.

[125] Atkinson J., Epand R. F., Epand R. M. (2008). Tocopherols and tocotrienols in membranes: a critical review. *Free Radical Biology & Medicine*.

[126] Ambrogini P., Torquato P., Bartolini D. (2019). Excitotoxicity, neuroinflammation and oxidant stress as molecular bases of epileptogenesis and epilepsy-derived neurodegeneration: the role of vitamin E. *Biochimica et Biophysica Acta (BBA) - Molecular Basis of Disease*.

[127] Franzoni F., Scarfò G., Guidotti S., Fusi J., Asomov M., Pruneti C. (2021). Oxidative stress and cognitive decline: the neuroprotective role of natural antioxidants. *Frontiers in Neuroscience*.

[128] Repici M., Mariani J., Borsello T. (2007). Neuronal death and neuroprotection: a review. *Methods in Molecular Biology*.

[129] Rahman M., Islam M., Rahman F. (2022). Emerging promise of computational techniques in anti-cancer research: at a glance. *Bioengineering*.

[130] Khodorova A., Nicol G. D., Strichartz G. (2017). The TrkA receptor mediates experimental thermal hyperalgesia produced by nerve growth factor: Modulation by the p75 neurotrophin receptor. *Neuroscience*.

[131] Fernández A., Méndez M., Santis O. (2020). *SUMOylation regulates protein cargo in astrocyte-derived small extracellular vesicles*.

[132] Sharma D., Sethi P., Hussain E., Singh R. (2009). Curcumin counteracts the aluminium-induced ageing-related alterations in oxidative stress, Na+, K+ ATPase and protein kinase C in adult and old rat brain regions. *Biogerontology*.

[133] Shen L. R., Xiao F., Yuan P. (2013). Curcumin-supplemented diets increase superoxide dismutase activity and mean lifespan in drosophila. *Age*.

[134] Rauf A., Abu-Izneid T., Khalil A. A. (2021). Berberine as a potential anticancer agent: a comprehensive review. *Molecules*.

[135] Zeisel S. H. (2004). Nutritional importance of choline for brain development. *Journal of the American College of Nutrition*.

[136] Zeisel S. H., Da Costa K. A. (2009). Choline: an essential nutrient for public health. *Nutrition Reviews*.

[137] Mehta A. K., Arora N., Gaur S. N., Singh B. P. (2009). Choline supplementation reduces oxidative stress in mouse model of allergic airway disease. *European Journal of Clinical Investigation*.

[138] Rahman M. M., Bibi S., Rahaman M. S. (2022). Natural therapeutics and nutraceuticals for lung diseases: traditional significance, phytochemistry, and pharmacology. *Biomedicine & Pharmacotherapy*.

[139] Alsherbiny M., Li C. (2019). Medicinal cannabis—potential drug interactions. *Medicine*.

[140] deShazo R. D., Parker S. B., Williams D. (2019). Marijuana's Effects on Brain Structure and Function: What Do We Know and What Should We Do? A Brief Review and Commentary. *The American Journal of Medicine*.

[141] Bonn-Miller M. O., Loflin M. J. E., Thomas B. F., Marcu J. P., Hyke T., Vandrey R. (2017). Labeling accuracy of cannabidiol extracts sold online. *JAMA*.

[142] Leos-Toro C., Fong G. T., Meyer S. B., Hammond D. (2020). Cannabis labelling and consumer understanding of THC levels and serving sizes. *Drug and Alcohol Dependence*.

[143] Dumont M., Beal M. F. (2011). Neuroprotective strategies involving ROS in Alzheimer disease. *Free Radical Biology & Medicine*.

[144] Orhan I., Aslan S., Kartal M., Şener B., Başer K. H. C. (2008). Inhibitory effect of Turkish *Rosmarinus officinalis* L. on acetylcholinesterase and butyrylcholinesterase enzymes. *Food Chemistry*.

[145] Omri A. E. L., Han J., Ben Abdrabbah M., Isoda H. (2012). Down regulation effect of Rosmarinus officinalis polyphenols on cellular stress proteins in rat pheochromocytoma PC12 cells. *Cytotechnology*.

[146] Erickson M. A., Banks W. A. (2013). Blood–brain barrier dysfunction as a cause and consequence of Alzheimer's disease. *Journal of Cerebral Blood Flow and Metabolism*.

[147] Huang R., Ke W., Han L. (2009). Brain-targeting mechanisms of lactoferrin-modified DNA-loaded nanoparticles. *Journal of Cerebral Blood Flow and Metabolism*.

[148] Sharma R., Kuca K., Nepovimova E., Kabra A., Rao M. M., Prajapati P. K. (2019). Traditional Ayurvedic and herbal remedies for Alzheimer’s disease: from bench to bedside. *Expert Review of Neurotherapeutics*.

[149] Mourtas S., Canovi M., Zona C. (2011). Curcumin-decorated nanoliposomes with very high affinity for amyloid-*β*1-42 peptide. *Biomaterials*.

[150] Wilson B., Selvam J., Mukundan G. K., Premakumari K. B., Jenita J. L. (2020). Albumin nanoparticles coated with polysorbate 80 for the targeted delivery of antiepileptic drug levetiracetam into the brain. *Drug Delivery and Translational Research*.

[151] Islam F., Bibi S., Meem A. F. K. (2021). Natural bioactive molecules: an alternative approach to the treatment and control of covid-19. *International Journal of Molecular Sciences*.

[152] Rahman M. M., Mim S. A., Tumpa M. A. (2022). Exploring the management approaches of cytokines including viral infection and neuroinflammation for neurological disorders. *Cytokine*.

[153] Derakhshankhah H., Hajipour M. J., Barzegari E. (2016). Zeolite nanoparticles inhibit A*β*-fibrinogen interaction and formation of a consequent abnormal structural clot. *ACS Applied Materials & Interfaces*.

[154] Krol S., Macrez R., Docagne F. (2013). Therapeutic benefits from nanoparticles: the potential significance of nanoscience in diseases with compromise to the blood brain barrier. *Chemical Reviews*.

[155] Huang M., Gu X., Gao X. (2018). Nanotherapeutic strategies for the treatment of neurodegenerative diseases. *Brain Targeted Drug Delivery System*.

[156] Franco R. (2021). Science plus technology to address challenges in determining the efficacy of neuroprotective/neurorestorative therapies. *Exploration of Neuroprotective Therapy*.

[157] Fischer J. R., Finnell J. (2006). Challenges and opportunities. *Resource Engineering and Technology for Sustainable World*.

[158] Panda P., Verma H. K., Lakkakula S. (2022). Biomarkers of oxidative stress tethered to cardiovascular diseases. *Oxidative Medicine and Cellular Longevity*.

[159] Vassalle C., Maltinti M., Sabatino L. (2020). Targeting oxidative stress for disease prevention and therapy: where do we stand, and where do we go from here. *Molecules*.

[160] Pandya R. S., Zhu H., Li W., Bowser R., Friedlander R. M., Wang X. (2013). Therapeutic neuroprotective agents for amyotrophic lateral sclerosis. *Cellular and Molecular Life Sciences*.

[161] Martin L. J., Liu Z. (2004). Opportunities for neuroprotection in ALS using cell death mechanism rationales. *Drug Discovery Today: Disease Models*.

[162] Werner C., Engelhard K. (2007). Pathophysiology of traumatic brain injury. *British Journal of Anaesthesia*.

[163] Valente S. M., Fisher D. (2011). Traumatic brain injury. *The Journal for Nurse Practitioners*.

[164] Torrey E. F. (1987). Hope through research. *New Directions for Mental Health Services*.

[165] Sharma R., Kakodkar P., Kabra A., Prajapati P. K. (2022). Golden Ager Chyawanprash with Meager evidential base from human clinical trials. *Evidence-based Complementary and Alternative Medicine*.

[166] Poddar M. K., Chakraborty A., Banerjee S. (2021). Neurodegeneration: diagnosis, Prevention, and Therapy. *Oxidoreductase*.

[167] Treglia G., Cason E., Giordano A. (2012). Diagnostic performance of myocardial innervation imaging using MIBG scintigraphy in differential diagnosis between dementia with Lewy bodies and other dementias: a systematic review and a meta-analysis. *Journal of Neuroimaging*.

[168] Shimizu S., Hirose D., Hatanaka H. (2018). Role of neuroimaging as a biomarker for neurodegenerative diseases. *Frontiers in Neurology*.

[169] Cruz L., Urbanc B., Inglis A., Rosene D. L., Stanley H. E. (2008). Generating a model of the three-dimensional spatial distribution of neurons using density maps. *NeuroImage*.

[170] Cruz L., Buldyrev S. V., Peng S. (2005). A statistically based density map method for identification and quantification of regional differences in microcolumnarity in the monkey brain. *Journal of Neuroscience Methods*.

[171] Buldyrev S. V., Cruz L., Gomez-Isla T. (2000). Description of microcolumnar ensembles in association cortex and their disruption in Alzheimer and Lewy body dementias. *Proceedings of the National Academy of Sciences of the United States of America*.

[172] Chance S. A., Clover L., Cousijn H., Currah L., Pettingill R., Esiri M. M. (2011). Microanatomical correlates of cognitive ability and decline: normal ageing, MCI, and Alzheimer's disease. *Cerebral Cortex*.

[173] Apostolova L. G., Thompson P. M. (2007). Brain mapping as a tool to study neurodegeneration. *Neurotherapeutics*.

[174] Kang X., Lewin H. C., Miranda R. (2000). Comparative localization of myocardial ischemia by exercise electrocardiography and myocardial perfusion SPECT. *Journal of Nuclear Cardiology*.

[175] Gee J. C., Thompson P. M. (2007). Guest editorial: special issue on computational neuroanatomy. *IEEE Transactions on Medical Imaging*.

[176] Thompson P. M., Woods R. P., Mega M. S., Toga A. W. (2000). Mathematical/computational challenges in creating deformable and probabilistic atlases of the human brain. *Human Brain Mapping*.

[177] Chen Y. W., Gurol M. E., Rosand J. (2006). Progression of white matter lesions and hemorrhages in cerebral amyloid angiopathy. *Neurology*.

